# Evaluation of Quality of Life of Those Living near a Wind Farm

**DOI:** 10.3390/ijerph120606066

**Published:** 2015-05-29

**Authors:** Bożena Mroczek, Joanna Banaś, Małgorzata Machowska-Szewczyk, Donata Kurpas

**Affiliations:** 1 Department of Humanities in Medicine, Faculty of Health Sciences, Pomeranian Medical University, 11 Chlapowskiego St., 70-204 Szczecin, Poland; E-Mail: b_mroczek@data.pl; 2 Faculty of Computer Science and Information Technology, West Pomeranian University of Technology, Szczecin Wydział Informatyki, 41 Zolnierska St., 71-210 Szczecin, Poland; E-Mails: jbanas@wi.zut.edu.pl (J.B.); mmachowska@wi.zut.edu.pl (M.M.-S.); 3 Department of Family Medicine, Wroclaw Medical University, 1 Syrokomli St., 51-141 Wroclaw, Poland; 4 Opole Medical School, 68 Katowicka St., 45-060 Opole, Poland

**Keywords:** quality of life, wind farm, correspondence analysis, SF-36v2

## Abstract

*Objectives*: Health-related quality of life (HRQoL) can serve as a multidimensional means of evaluating the relationship between the presence of wind turbines in residential areas and their consequence for health. The purpose of this study was to determine whether a relationship exists between the presence of wind farms at different stages of development and the HRQoL of people living in their vicinity in Poland. *Method*: The instruments employed in this study were the SF-36v2, a questionnaire measuring self-reported health problems, and a sociodemographic questionnaire. The study involved 1277 people who lived within 2 km from a wind turbine. *Results*: The highest overall QoL scores were obtained by respondents living the closest to wind turbines. The mental health, role emotional, and social functioning scores were significantly higher among respondents living near wind farms and wind-farm construction sites than among those living close to locations where wind farms were planned but where construction had not yet begun. Positive correlations were found between physical and mental component scores and reactions to the news of plans to construct a wind farm. Significant differences in physical and mental component scores were observed between residents who reacted calmly and those who responded with apprehension. Residents who expected the improvement of their financial standing as a result of the wind farm assessed their general health higher than those who did not expect to receive any economic benefits. The lowest QoL scores corresponded to frequent headaches, stomach aches, and back pain over the previous three months, as well as recurrent problems with falling asleep, anxiety, and a lack of acceptance of the project. *Conclusion*: The lowest overall QoL and general health scores were noted among residents of places where wind-farm developments were either at the stage of planning or under construction. In order to find ways of reducing environmental stress and its adverse effects on health, it is necessary to conduct research on residents of places where a wind farm is either planned or under construction, or has just been completed.

## 1. Introduction

The measurement of health-related quality of life (HRQoL) can serve as an alternative way to monitor the relationship between the presence of wind turbines and health problems experienced by people living in their vicinity. According to the available studies, those living near wind farms suffer from symptoms such as vertigo, headaches, sleep disorders, and irritation [1,2,3,4]. They also report exacerbation of diseases, especially of hypertension, diabetes, migraine, and heart disease [5,6]. The literature on the subject is focused mainly on health complaints in the form of nuisances, attitudes towards wind energy, visual effects, and the feeling of stress [7]. No scientific evidence has been found so far in favor of the influence of turbines (in particular, of their noise) on health [1,2].

Most studies that have been conducted so far concern the effects of wind-farm noise on human health and quality of life. One major conclusion is that the noise and visual effects are related to sleep disorders [4,7,8,9,10,11,12,13,14]. Although irritation is not regarded by the WHO as a disease entity, it may contribute to the development of other disorders [15].

Another interesting piece of research analyzed the quality of life (QoL) of people living near wind farms in relation to their objective living conditions, current life situation, health status, and social factors [16,17].

Nissenbaum *et al.* measured QoL using the Short Form Health Survey Version 2 (SF*-*36v2). Their study involved a group of 38 adult residents of places located 375–1400 m from two wind farms in Maine, USA (the study group), and a group of 41 subjects living at a distance of 3–7 km from a wind farm (the control group). Both the quality of life within the SF-36 mental health (MH) domain and the quality of sleep were considerably lower in the study group than in the control group [3].

In 2009, a study of the HRQoL, as measured by the SF*-*36v2, was carried out among residents of Zagórze and Jagniątkowo in Poland. These localities, in the commune of Wolin in the province of West Pomeranian, are located near wind farms [18]. In year 2010, the study was repeated in the same places, using the same questionnaire, with a group of 82 subjects [19]. It was demonstrated that the presence of the wind farms did not decrease the QoL of the people living in these places. The residents assessed their QoL higher than in the study from 2009. The health status of 66% of the respondents did not change in comparison to the previous year, although some of them reported occasional problems with concentration, anger, and anxiety.

Considering the above, the purpose of this study was to determine whether there exists a relationship between the presence of wind farms at different stages of development and the QoL of people living in their vicinity in Poland.

## 2. Materials and Method

### 2.1. Selection of the Area and Number of Respondents for the Study

The number of respondents in the study group was statistically determined using a two-stage sampling method, based on stratification into the presence of operational wind farms and those planned or under construction in 2010. From the 16 provinces (voivodeships) constituting Poland, we selected the five provinces with the highest number of wind farms in 2010, where the projects were under construction or in the process of public consultation [20]. Next, localities in the vicinity of the wind farms in these regions were selected.

A random sampling method was employed to select respondents who met the following criteria: age above 18 years and place of residence within 2 km from wind-farm projects at various stages of development. We only invited adults entitled to vote on changes in the local environment. The questionnaires were filled out by one person per household. In those selected localities that had populations of up to 100 residents, all adults were invited to take part in the study. The study was anonymous and conducted using the door-to-door method. Each participant was asked to give his or her consent to participate in the study. Some of those invited refused to participate: the overall refusal rate was about 15 percent.

#### Participants

The study involved a group of 1277 residents of places located near wind farms in Poland.

Distance between the wind farm and the place of residence. The study group was divided into five subgroups on the basis of the approximate distance between the place of residence and the nearest turbines in the wind farms at various stages of investment. Some 17.23% (220) lived within 700 m of the nearest turbine, 21.85% (279) within 701–1000 m, 17.31% (221) within 1001–1500 m, and 33.2% (424) within 1500–2000 m. One in ten respondents (10.41%, 133) was unable to estimate the distance (though according to the developers, this could not have exceeded 2 km). Overall, 101 (7.91% of all participants) lived near planned wind farms or wind farms under construction, while 32 (2.51%) lived near wind turbines. Problems with estimating distance were associated with the stage of development: people who lived near planned wind farms or wind-farm construction sites were unable to estimate the distance (Chi^2^ = 101.76, *p* = 0.0001).

Stage of project development*.* Some 40% (511) of the respondents lived near to completed wind farms, 33% (421) near to wind-farm construction sites, and 27% (345) near planned wind-farm projects currently in the process of public consultation. The project was approved by the Bioethical Commission of the Pomeranian Medical University in Szczecin, Poland [KB-0012/83/10].

Women constituted 55.05% (703) of the people surveyed, and men made up 44.95% (574). The mean age was 45.54 ± 16.1 years. The majority of the respondents were employed (51.60%), and 8.46% earned their living as farmers only. The sociodemographic data are shown in Table 1.

**Table 1:** Sociodemographic data.

Variables			*n* = 1277
Gender	Women	*n* (%)	703 (55.05)
	Men	*n* (%)	574 (44.95)
Age (for whole group)		*n*	1277
		M ± SD	45.54 ± 16.10
		Me	45.5
		Min, Max	18–90
Age	Women	*n*	703 (55.05)
		M ± SD	46.2 ± 16.10
		Me	46
		Min, Max	18–94
	Men	*n*	574 (44.95)
		M ± SD	44.7 ± 16.00
		Me	45
		Min, Max	18–85
Education	Primary	*n* (%)	332 (26.00)
	Vocational	*n* (%)	440 (31.30)
	Secondary	*n* (%)	397 (31.09)
	Higher	*n* (%)	139 (10.90)
	No data	*n* (%)	9 (0.70)
Place of residence	Villages	*n* (%)	1277 (100.00)
Professional activity	Employed	*n* (%)	659 (51.60)
	Disability pension—Retirement pension	*n* (%)	189 (14.80)
	Unemployed	*n* (%)	335 (26.23)
	Student	*n* (%)	80 (6.26)
	No data	*n* (%)	14 (1.09)

### 2.2. Survey-Based Study

The influence of the wind farms in their various stages of development on the residents’ QoL was assessed using the Polish version of the SF-36v2 questionnaire (SF-36v2™ Health Survey). A license to use this instrument was obtained from the Office of Grants and Scholarly Research (OGSR). SF-36^®^ is a registered trademark of Medical Outcomes Trust (SF-36v2™ Acute Interview Script, Poland (Polish). The survey was conducted by well-prepared pollsters, in whose presence respondents completed the questionnaire. Some respondents asked the pollsters to read out the questions and to mark the answers that they indicated.

The SF-36v2 consists of 36 questions divided into eight subscales measuring eight areas of human functioning (Table 2). The score on the Likert scale for each of these areas varies from 0 to 100, with 0 denoting the worst possible and 100 the best possible health status. The SF-36v2 was the questionnaire of choice because of its high sensitivity to changes in health status, its repeatability, and the availability of population normative data, which make this instrument useful in comparative analysis [3,18,19].

**Table 2:** SF-36 domains with description.

Abbreviation	Domain	Domain Description
**PF**	Physical Functioning	Limitations in physical activities because of health problems
**RP**	Role Physical	Limitations in usual role activities because of physical health problems
**BP**	Bodily Pain	Intensity of bodily pain or discomfort
**GH**	General Health	General health perceptions
**VT**	Vitality	Energy and fatigue
**SF**	Social Functioning	Limitations in social activities due to physical or emotional problems
**RE**	Role Emotional	Limitations in usual role activities because of emotional problems
**MH**	Mental Health	Psychological distress and well-being

The QoL of the respondents is represented by a synthetic variable, which is an arithmetic mean of the QoL scores from the eight SF-36 domains (physical functioning, role physical, bodily pain, general health, vitality, social functioning, role emotional, and mental health). The scores were obtained on the basis of Likert-type questions with numerically coded answers. The SF-36 questionnaire allowed us to assess QoL in terms of the physical component (PCS) and the mental component (MCS). A low score reflects a negative subjective perception of health, resulting from pain and disability. A high score, on the other hand, suggests good health status and high QoL. According to Ware *et al.*, the scores for overall QoL and general health are more stable than the scores in the other domains. These authors believe that it is more useful to base the overall QoL assessment on mental and physical component scores than the scores in the specific SF-36 domains [21,22]. In our study, the reliability of SF-36v2 was assessed using Cronbach’s alpha coefficient for measuring the internal coherence of the scale [23]. The results obtained suggest the high internal coherence of the SF-36 domains when the value of Cronbach’s alpha exceeds 0.95 for physical functioning and 0.76 for mental health. The lowest coherence coefficient (0.71) was obtained for social functioning. The SF-36v2 questionnaire applied to the study of people living near wind farms at different stages of wind-farm development is characterized by acceptable reliability and accuracy. We also employed three questionnaires of our own design: one for measuring self-reported health problems; one to collect information concerning sociodemographic data, risky health behaviours, and chronic diseases; and one to evaluate the social acceptance of the investment in wind energy.

In the study, we took into consideration 12 health problems associated with feelings of stress and irritation caused by the wind-farm projects in residential areas. The respondents marked the severity of their symptoms on a scale divided into two categories: often and seldom. The health problems included in the study covered the most common chronic diseases. Respondents marked diseases that they suffered from (yes/no) [14]. Risky drinking was evaluated on the basis of responses given to questions concerning the frequency of alcohol consumption (once a month or rarer, once a week, more than once a week, only occasionally, I have not drunk alcohol during the last year) [24]. Questions concerning smoking were taken from the Fagerstrom questionnaire for nicotine dependence [25] and answered using the following scale: I smoke regularly (nicotine addiction), I smoke occasionally, I have given up smoking (broken habit), and I have never smoked. In response to the question concerning their first reaction to the news of the planned wind farm, respondents ticked the box of one of the following answers: I was calm; I do not care; I did not believe it; I was nervous; I do not remember my reaction.

### 2.3. Statistical Analysis

A generalized linear model (GLZ) was applied to determine which factor had the most profound contribution to the QoL level of people living in wind farm areas. The authors took into consideration the degrees of freedom (df), the significance of the effects included in the model (Wald statistics), a log-likelihood ratio statistic, the level of significance, the effect level, the estimate, the standard deviation, the confidence interval, and the odds ratio.

The authors additionally employed correspondence analysis, which is a method of multidimensional statistical analysis based on diagnostic variables describing quality of life [26]. All the variable categories were accepted as objects, and the projection coordinates of each category were treated as variants. The results of the correspondence analysis are presented using Ward’s method [27]. The authors accepted the following set of variable categories and their variants:

(1) *QoL*: quality of life (0; 25; 50; 75; 100).
(2) *Age* (below 30; 31–40; 41–50; 51–60; over 61).
(3) *Gender* (W: women; M: men).
(4) *Edu*: education (P: primary; V: vocational; S: secondary; H: higher).
(5) *Empl:* employment (E: employed; U: unemployed; S: student; P: pensioner).
(6) *Dist_WF:* distance between residence and wind farms (below 700 m; up to 700 m; 701–1000 m; 1001–1500 m; 1501–2000 m; unkn: I do not know, but no more than 2 km),
(7) *WEi*: wind farm status (UC: under construction; P: planned; WF: completed; unkn: I don’t know),
(8) *Alc*: alcohol consumption (S: seldom or never; O: often),
(9) *Cig:* smoking (DS: I don’t smoke; S: I smoke regularly),
(10) *Compl_P:* frequency of headaches, stomach aches, or back pain during the last six months (S: seldom; O: often),
(11) *Compl_Ir:* frequency of feeling depressed, irritated, angry, or nervous during the last three months (S: seldom; O: often),
(12) *Compl_Sl:* frequency of having trouble falling asleep or being anxious during the last three months (S: seldom; O: often),
(13) *Compl_F:* frequency of feeling very tired or exhausted during the last 3 months (S: seldom; O: often),
(14) *ChroD:* being treated for chronic disease (Y: yes; N: no).

Gender and Compl_Ir were shown to be unrelated to QoL, and thus excluded from the multidimensional correspondence analysis*.* The authors obtained a Burt matrix, reflecting the dimensions of the real coexistence space of 27 variants.

Statistical analysis was performed using Statistica 10 PL software. The level of significance was set at *p* ≤ 0.05. The coexistence of QoL (as a dependent variable) and the other variables analyzed in this study was assessed by means of multidimensional correspondence analysis [28]. The authors employed Microsoft Excel 2007 and Statistica 10 PL software in the analysis.

## 3. Results

The most common diseases in the study group were hypertension (26.62%, 340), rheumatism (14.17%, 181), coronary heart disease (12.84%, 164), and diabetes (11.82%, 151). The coexistence of two or more chronic diseases was noted in 24.51% (314) of the respondents: in women, this was twice as common as in men (44.6%, 314 *vs.* 22.2%, 127) (*p* < 0.001).

Health problems that occurred almost every day included headaches (41.89%, 535), nervousness (41.82%, 522), back pain (39.57%, 497), and severe fatigue (36.27%, 432). Anger was reported by 30.61% (391), irritation by 29.83% (381), sleep problems by 28.34% (362), and anxiety sleep problems by 15.58% (194). There were statistically significant relationships between the levels of anxiety, nervousness and anger (often—almost every day and several times a month) and the presence of wind farms, as well as between the levels of anxiety and anger and the presence of the wind-farm development in the planning stage (Table 3). The average BMI (Body Mass Index) value was 26.61 ± 5.54. BMI values over 30 kg/m^2^ were noted in 21.20% (266), and in more women than men (14.79%, 104 *vs.* 12.93%, 74). Smokers constituted 61.17% (526), and risky drinkers made up 32.89% (420) of the respondents.

### 3.1. Quality of Life of People Living in the Vicinity of Wind Farms

The average QoL score in the study group was 61.85 ± 23.40. The highest average QoL score was obtained for physical functioning (76.12 ± 27.92, CI 74.51–77.58) and the lowest for general health (55.33 ± 24.04, CI 53.96–56.61). Differences in the QoL scores in the individual SF-36 subscales (determined using Kendall’s coefficient of concordance) were statistically significant for *p* = 0.0001, W = 0.098 (Table 4). There was a significant relationship between the QoL scores in all eight domains, age, and level of education. Older respondents and those with lower levels of education (primary and vocational only) assessed their QoL as lower. The perception of changes in health status also significantly differed depending on age and level of education.

### 3.2. The Influence of Environmental Stress Factors on Assessment of Quality of Life

Environmental stress factors included the distance from the residence to the nearest wind turbine, as estimated by the respondents, the presence of a development, and its status (planned, under construction, or finished).

#### 3.2.1. The Influence of Distance

The highest overall QoL scores were obtained by respondents living closest to wind turbines (up to 700 m), and the lowest by those living at a distance of 1501–2000 m (H = 32.25, *p* = 0.001). Respondents living 1501–2000 m from a wind farm had significantly lower QoL scores for general health (H = 19.93, *p* < 0.001), vitality (H = 20.04, *p* < 0.001), social functioning (H = 113.35, *p* < 0.0001), and mental health (H = 60.74, *p* < 0.001) than those living closest (up to 700 m). The lowest average QoL scores for role physical (50.56 ± 41.68), general health (51.96 ± 25.20), and role emotional (52.13 ± 43.49) were noted among those respondents who were unable to estimate the distance between their house and the wind farm.

**Table 3:** Analysis of LOGIT regression function of anxiety level and the levels of irritation, nervousness, and anger with regard to the presence of wind farms and distances between houses and wind turbines.

Variable		Anxiety						Irritation						Nervousness						Anger					
**Level**		**Score**	**SD**	**the Wald Statistic**	**CI +95%**	**CI −95%**	***p***	**Score**	**SD**	**the Wald Statistic**	**CI +95%**	**CI −95%**	***p***	**Score**	**SD**	**the Wald Statistic**	**CI +95%**	**CI −95%**	***p***	**Score**	**SD**	**the Wald Statistic**	**CI +95%**	**CI −95%**	***p***
Stage of investment *	**1**	**0.47**	**0.11**	**16.74**	**0.24**	**0.69**	**0.000**	−0.07	0.11	0.52	−0.28	0.13	0.70	**−0.52**	**0.10**	**24.67**	**−0.73**	**−0.31**	**0.000**	**0.62**	**0.11**	**32.15**	**0.40**	**0.83**	**0.000**
	**2**	**−0.25**	**0.11**	**5.59**	**−0.46**	**−0.04**	**0.018**	0.04	0.10	0.14	−0.16	0.24	0.710	**0.31**	**0.10**	**9.57**	**0.15**	**0.51**	**0.002**	**−0.40**	**0.10**	**15.82**	**−0.60**	**−0.20**	**0.000**
	**3**	**−0.41**	**0.14**	**0.03**	**−0.68**	**−0.12**	**0.004**	0.06	0.13	0.19	−0.20	0.31	0.661	0.15	0.19	1.30	−0.11	0.40	0.253	−0.24	0.13	3.23	−0.49	0.02	0.072
Distance from house to wind farms in meters **	**O1**	**0.70**	**0.20**	**12.16**	**0.31**	**1.10**	**0.000**	−0.12	0.16	0.52	−0.44	0.20	0.470	−0.22	0.16	1.82	−0.55	0.10	0.178	**0.36**	**0.16**	**4.63**	**0.03**	**0.68**	**0.031**
	**O2**	−0.08	0.14	0.33	−0.34	0.19	0.564	−0.11	0.13	0.70	−0.36	0.14	0.403	0.09	0.13	0.56	−0.16	0.35	0.455	0.03	0.13	0.04	−0.23	0.28	0.840
	**O3**	−0.25	0.14	2.86	−0.53	0.04	0.090	0.08	0.14	0.32	−0.20	0.36	0.571	0.19	0.14	1.77	−0.09	0.46	0.184	−0.24	0.14	2.92	−0.52	0.04	0.087
	**O4**	−0.07	0.11	0.38	−0.30	0.15	0.539	−0.03	0.11	0.08	−0.25	0.18	0.769	−0.06	0.11	0.26	−0.27	0.17	0.606	0.02	0.11	0.05	−0.19	0.24	0.828

* Investment: 1—completed; 2—under construction; 3—to be implemented; ** Distance: O1—below 700 m; O2—701–1000 m; O3—1001–1500 m; O4—1501–2000 m.

**Table 4:** Analysis of participants’ mean scores for quality of life in eight SF-36 scales.

SF-36 Scale *	X ± SD	CI −95%	CI +95%	N	W	*p*
Physical functioning	76.12 ± 27.92	74.51	77.58	1277	0.098	0.0001
Limitations in daily activities due to physical health (role physical)	59.87 ± 39.26	57.67	61.98	1276		
Bodily pain	63.70 ± 32.22	61.89	65.43	1277		
General health	55.33 ± 24.04	53.96	56.61	1277		
Vitality	58.23 ± 24.15	56.90	59.55	1277		
Social functioning	58.75 ± 36.32	56.75	60.74	1277		
Role emotional	62.73 ± 40.36	60.51	64.94	1276		
Mental health	60.13 ± 23.06	58.87	61.40	1276		

* Mini–Max = 0–100; W—Kendall’s coefficient of concordance; *p*—level of significance.

#### 3.2.2. The Influence of the Stage of Development on Assessment of Quality of Life

Regardless of the stage of development of the nearby wind farm, the QoL scores for physical functioning, role physical, and bodily pain did not significantly differ (all *p* > 0.05), whereas the scores for general health, mental health, role emotional, vitality, and social functioning were statistically significantly related to the stages of investment (all *p* < 0.05). Respondents living near construction sites and wind farms had significantly higher scores for role emotional than those living close to the planned investment and showing nonacceptance of the news (H = 16.341, *p* = 0.001).

There were differences in the scores for social functioning between people living near wind farms and those living near wind-farm construction sites (H = 17.72; *p* = 0.011), and between respondents living near wind farms and respondents who did not accept the development (H = 19.72, *p* = 0.0001). Respondents living near construction sites assessed their general health as being lower than those from places where the wind-farm development was in the planning stage (H = 8.37, *p* = 0.04). Differences in mental health assessment were noted between respondents who did not accept the development and those living near wind farms and construction sites (H = 12.46, *p* = 0.02). The vitality scores were significantly related to the presence of wind farms and nonacceptance of the development (H = 14.02, *p* = 0.003).

#### 3.2.3. Reactions to Learning of Wind-Farm Projects

The most common reactions to the news that a wind-farm was to be built were calmness (43.65%, 523) and indifference (21.28%, 255), followed by nervousness (9.69%, 115). Some 17.61% (211) did not remember their reactions. Significant differences in physical component scores (H = 3.34; *p* = 0.008) and mental component scores (H = 4.24; *p* = 0.001) were observed between those who reacted calmly and those who responded with nervousness. Mental component scores also differed between the respondents who reacted calmly to the news of the development and those who did not recall their own reactions (H = 3.11; *p* = 0.02).

### 3.3. Expectations of Benefits Associated with the Investment

Residents who derived real economic benefits by leasing their land for wind-farm construction constituted 8.65% (110) of the sample, and those who expected to be able to lease their land at the planning stage made up 21.78% (277). Residents who expected their financial standing to improve assessed their general health as higher than those who did not expect any changes in their economic situation (H = 8.285, *p* = 0.016).

Statistically significant differences in the scores for vitality (H = 8.259, *p* = 0.018), social functioning (H = 49.192, *p* = 0.0001), and mental health (H = 49.192, *p* = 0.0001) were observed between respondents who expected an offer to lease their land and those who did not lease their land. The scores for role emotional were lower among respondents who expected an offer to lease their land in the planning stage than among those who leased their lands (H = 10.230, *p* < 0.05).

### 3.4. Result of Correlations

When the results were statistically significant, a multiple regression of dependent variables was performed (on the eight separate SF-36 domains, physical component, and mental component). The strongest contributors to QoL scores in all the SF-36 domains, as well as in the physical and mental components, were age and somatic symptoms of stress, such as stomach ache, headache, and back pain (Table 5). Education, as a determinant of socioeconomic status, had the strongest influence on QoL scores in the physical component, and in the domains of physical functioning and bodily pain. The higher the level of education, the higher the QoL scores in these domains.

The stage of development and proximity from home of the wind farm were the strongest contributors to the feeling of anxiety (W = 27.82, *p* = 0.0001 *vs.* W = 12.27, *p* = 0.01). Irritation (OR = 1.49), anxiety (OR = 0.66), anger (OR = 0.87), and nervousness (OR = 0.80) occurred more frequently in respondents who lived closer to the developments than in those living about 2 km from the development. People living close to wind-farm construction sites feel nervous (W = 32.56, *p* = 0.0001) and angry (W = 46.01, *p* = 0.0001).

**Figure 1:**
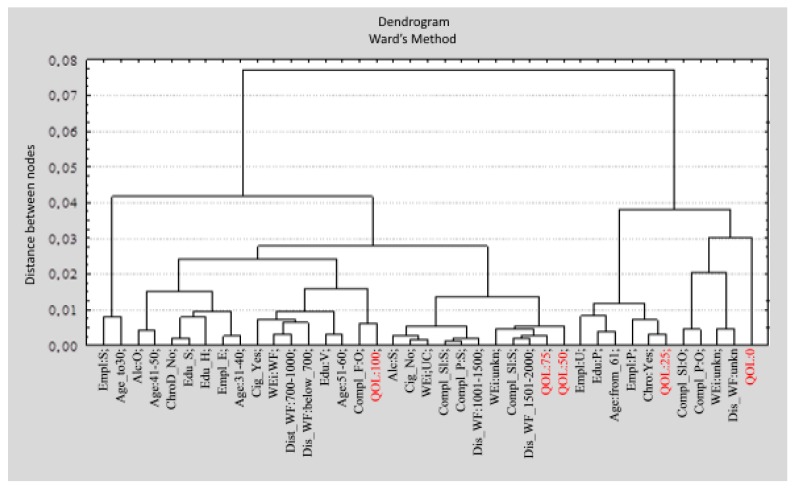
Diagram showing the division into variable categories performed by means of Ward’s method (source: authors’ own design). Legend: **Class I:** Empl_S; Age_to 30; **Class II-1:** Alc_O; Age_41-50; ChroD_No; Edu_S; Edu_H; Empl_E; Age_31-40; Cig_Yes; WEi_WF; Dist_WF_700-1000; Dis_WF_below_700; Edu_V; Age_51-60; Compl_F_O; QOL_100; **Class II-1:** Alc_S; Cig_No; WEi_UC; Compl_Sl_S; Compl_P_S; Dis_WF_1001-1500; WEi_unkn; Compl_Sl_S; Dis_WF_1501-2000; QOL_75; QOL_50; **Class III:** Empl_U; Edu_P; Age_from_61; Empl_P; Chro_Yes; QOL_25; **Class IV:** Compl_Sl_O; Compl_P_O; WEi_unkn; Dis_WF_unkn; QOL_0.

The results of the correspondence analysis complement the available data concerning the QoL of people living in the vicinity of wind farms (Figure 1).

A division into four classes was proposed. **The first class** indicates the connection between being a student and age up to 30 years. There are two clusters in **the second class**. The highest QoL scores are related to a lack of chronic diseases, but also to a frequent feeling of fatigue, professional activity at ages over 30, education higher than primary, smoking cigarettes, frequent alcohol consumption, and the presence of wind farms within a kilometer from the residence. Average and high QoL scores are associated with the rare occurrence of the health problems investigated in this study, abstinence from alcohol and nicotine, and wind farms being more than one kilometer from the residence. Low QoL scores (**third class**) are related to age (over 60 years), professional inactivity (unemployment or pension), primary education only, and chronic diseases. The lowest QoL scores are linked to frequent headaches, stomach aches, and back pain over the previous three months, recurrent difficulty falling asleep, anxiety during the previous three months, and a lack of acceptance of development near the place of residence (**fourth class**).

**Table 5:** Analysis of multiple regression results for determination of factors having the strongest impact on quality of life in SF-36 eight domains as well as physical component and mental component.

SF-36 Scale		Sociodemographic Data			Diseases								Risky Health Behavior	
		* Age	Professional Activity	Education	Arthritis	Hypertension	Heart Disease	Pulmonary Disease	Alcohol Dependency	Diabetes Mellitus	Other Diseases	Cancer	Smoking	Alcohol Consumption
**PCS**	β	−0.286	0.050	0.068	−0.072	−0.056			0.059		−0.055		0.53	−0.108
	*p*	0.001	0.04	0.01	0.01	0.05			0.05		0.02		0.02	0.001
**MCS**	β	−0.184				0.065								
	*p*	0.001				0.03								
**PF**	β	−0.323	−0.073	0.073								0.078		
	*p*	0.001	0.01	0.01								0.01		
**RP**	β	−0.217			−0.062	−0.082					−0.061			
	*p*	0.001			0.03	0.01					0.02			
**BP**	β	−0.207		0.065	−0.069				0.069					
	*p*	0.001		0.02	0.01				0.032					
**GH**	β	−0.260	−0.053		−0.118						−0.084			−0.106
	*p*	0.001	0.02								0.001			0.001
**VT**	β	−0.203			−0.058					0.064	−0.054			−0.087
	*p*	0.001			0.03					0.019	0.03			0.001
**SF**	β	−0.168												−0.131
	*p*	0.001												0.001
**RE**	β	−0.175	−0.064			−0.090					−0.072			
	*p*	0.001	0.001			0.01					0.01			
**MH**	β	−0.079					0.065	−0.058						−0.075
	*p*	0.01					0.03							0.001
**SF-36 Scale**		**Health Problems**											**Stressors**	
		**Headaches**	**Stomach aches**	**Backaches**	**Depression**	**Anxiety**	**Irritability**	**Fatigue**	**Exhaustion**	**Anger**	**Uneasiness**	**Insomnia**	**Distance of WF**	**Stage of Investment**
**PCS**	β	−0.131	−0.102	−0.168		−0.102		0.075	0.115					
	*p*	0.001	0.001	0.001		0.001		0.01	0.001					
**MCS**	β	−0.175	−0.209	−0.098	−0.059	−0.108							−0.109	
	*p*	0.001	0.001	0.01	0.046	0.001							0.001	
**PF**	β	−0.122	0.094	−0.097				0.062	0.015					
	*p*	0.001	0.001	0.001				0.04	0.001					
**RP**	β		0.095	−0.145	−0.068	−0.114		0.069	0.081		0.08		−0.064	
	*p*	0.001		0.001	0.03	0.001		0.04	0.01		0.02		0.02	
**BP**	β	−0.164	0.073	−0.186		−0.102			0.079					
	*p*	0.001		0.001		0.001			0.001					
**GH**	β	−0.161	−0.123	−0.125		−0.124			−0.055			−0.06		−0.053
	*p*	0.001	0.001	0.001		0.001			0.04			0.02		0.03
**VT**	β	−0.200	−0.202	−0.109		−0.104		0.061		−0.062				−0.069
	*p*	0.001	0.001	0.001		0.001		0.04		0.04				0.01
**SF**	β	−0.177	−0.211										0.147	
	*p*	0.001	0.001										0.001	
**RE**	β		−0.099	−0.107		−0.146			0.107				0.064	
	*p*		0.001	0.001		0.001			0.001				0.001	
**MH**	β	0.217	0.227	0.078	0.101	−0.125		0.065		0.088				
	*p*	0.001	0.001		0.001	0.001		0.001		0.01				

* Only those factors that demonstrated statistically significant impact on SF-36 quality of life scores were included.

## 4. Discussion

New developments in the living environment are a kind of environmental stress factor that can affect both human health and quality of life [1,2,3,6,12,13,14]. The appearance of a new building and the related social reactions may violate certain standards that are necessary to most members of the particular community [29,30]. Another argument for research on the influence of wind turbines on human health is the growing number of people exposed to their operation. A multitude of complaints about the worsening of health status and the occurrence of diseases whose onset or exacerbation is attributed to wind turbines, show that more extensive research in this field is necessary.

It was assumed in this study that the QoL of people living in the vicinity of wind farms is significantly influenced by stress factors related to such surroundings, including the stage of development of the farm, the distance between residence and the wind turbines, and the acceptance of wind farms. The results of this study do not indicate that chronic diseases and risky health behaviors decrease the quality of life, whereas age, education, and professional activity do.

Age is the sociodemographic factor that had the greatest effect on QoL in the multiple regression model. An older age corresponded with lower QoL scores in the physical component, the mental component, and in all SF-36 domains. Residents of wind-farm areas assessed their general health as the lowest of all SF-36 domains. Health status was usually assessed as average or good, hence the lower QoL scores [31]. The results obtained may be associated not only with the age of the respondents, but also with chronic diseases and other health problems. We cannot say that there is a clear relationship between the occurrence or exacerbation of diseases and living in a wind-farm area, since we did not ask the respondents about the moment of the onset or exacerbation of the disease.

The distance between housing and wind farms is a factor that has significant effects on HRQoL scores in both the mental component (MCS) and the domains of vitality, social functioning, role emotional, and role physical. Those respondents living closest to wind farms assessed their quality of life higher on all SF-36 domains than those living 1500–2000 m from the wind farm and those who were unable to estimate the distance. The group of respondents who were unable to estimate the distance included people living close to a planned development or a development under construction, which could contribute to the problems with estimating the distance. This can be explained by a cognitive theory: people living closest to wind farms had access to information that let them perceive the situation in terms of potential profit and loss. They regarded investment in wind energy as significant from the point of view of both their own and social interests [4,14,32,33]. The answer can be associated with low levels of education and the lack of acceptance of the development. The results can be also explained by the symptoms of environmental stress caused by factors such as the investment in wind energy, changes in the landscape, the distance between the wind farm and the home, fear for the value of the land, insufficient information about the development, contradictory information concerning the influence of wind farms on human health and the environment, a lack of acceptance of the investment, *etc*.

It was observed that lower scores for mental health correlated with an exacerbation of mental health problems, such as irritation, fatigue, exhaustion, and depression. Still, it cannot be unambiguously stated that these health problems resulted from the presence of the wind-farm project or from the distance between housing and the wind turbines. The lack of data concerning the occurrence of these problems, combined with the installation of wind turbines, hinders the interpretation of the results regarding the health problems reported by residents [2]. Living near to the wind-farm development is a strong contributor to the feeling of anxiety and irritation, but detailed analysis demonstrates that the stage of the development contributes even more strongly. It was assumed in this study that anxiety is a serious health problem that has an influence on the QoL level of people living in wind-farm areas. However, a population study conducted in New Zealand showed that anxiety affected 14.1% of respondents (*n* = 1000), which means that it is a feeling experienced by people every day, regardless of the factors that provoke it [34]. Patients attribute the occurrence of these symptoms to other factors, such as the influence of an electromagnetic field, and associate this factor with working wind turbines [34,35,36]. In light of these facts, it is possible that bouts of symptoms in our study were caused by environmental stress factors. It is necessary to perform a comparative analysis of the exposure to environmental stress factors, such as wind-energy development and living in big cities or near a freeway.

There was a strong relationship between the frequency of feeling anxious, nervous, or angry by the residents and all stages of investment (from planning and public consultation, through construction, to operation). Proximity to wind farms (a distance of less than 700 m) is a strong contributor to the feeling of anxiety and anger. It is worth mentioning that Laszlo 2012 recommends that research performed in an altered living environment be conducted both before and after the change [36]. Similar conclusions were drawn by Bakker *et al.* 2012 [37].

A very important aspect of the QoL of people living near wind turbines is their acceptance of wind farms. It has been observed that the degree of acceptance significantly correlates with whether individuals obtain payment for leasing the land, and with expectations of both personal benefits and subsidies for the community [38]. The observations of Pedersen and Persson Waye reveal that the noise generated by wind turbines is more bothersome for people who show negative attitudes towards such developments and is considerably less troublesome for those who derive economic benefit from it [39]. The degree of irritation noted in those who derived benefit from the wind farm was significantly lower than in those who did not gain in this way, even though they were exposed to comparable noise levels [4,8,9,11,13,39]. Similar results concerning QoL and acceptance of wind farms were obtained by Johanson and Laike [40]. According to these authors, a better understanding of the social factors, based on psychological theories, would allow us to realize why residents oppose the construction of wind turbines [4,8,9,34,37].

Both the outcomes of this study and the findings reported by other researchers show that health problems may be triggered by many factors, including lifestyle, health behaviors, diseases, and subjective expectations or attitudes towards wind energy projects. Irritation-related health problems may evoke other symptoms through the “nocebo” effect. This latter results from negative attitudes towards wind farms and the lack of acceptance of their presence in the nearby environment [4,41]. The nocebo effect may be also associated with the high sensitivity of some people to the influence of environmental factors. In such cases, health problems that occur may be attributed to the presence of wind turbines [2].

The available results imply that wind turbines, when located at a proper distance, do not exert any negative effects on human health [4,7,8,42].

## 5. Conclusions

(1) Age is the strongest contributor to QoL levels in wind-farm areas. It is possible that QoL is simultaneously influenced by several factors, such as chronic diseases and other health problems, adverse socioeconomic factors, and environmental stress factors.
(2) The lowest scores for overall QoL and general health are noted among residents of places where projects are in the planning or construction phase. In order to find ways to reduce environmental stress and its adverse effects on health, it is necessary to conduct research among the residents of places where a wind-farm project is either being planned or is under construction or has just been completed [2,34,43].
(3) The presence of wind farms near residential areas has no negative influence on the QoL of residents. The highest QoL levels are noted in places where wind farms at various stages of development are located within one kilometer from the residence.
